# High seroprevalence of echinococossis, schistosomiasis and toxoplasmosis among the populations in Babati and Monduli districts, Tanzania

**DOI:** 10.1186/s13071-014-0505-7

**Published:** 2014-11-12

**Authors:** Mohammad Behram Khan, Parthasarathy Sonaimuthu, Yee Ling Lau, Hesham M Al-Mekhlafi, Rohela Mahmud, Nicholas Kavana, Ayub Kassuku, Christopher Kasanga

**Affiliations:** Department of Parasitology, Faculty of Medicine, University of Malaya, Kuala Lumpur, Malaysia; Department of Veterinary Microbiology and Parasitology, Faculty of Veterinary Medicine, Sokoine University of Agriculture, Morogoro, Tanzania

**Keywords:** Echinococcosis, Schistosomiasis, Toxoplasmosis, Zoonosis, Seroprevalence, Tanzania

## Abstract

**Background:**

The neglected tropical diseases, echinococcosis, schistosomiasis and toxoplasmosis are all globally widespread zoonotic diseases with potentially harmful consequences. There is very limited data available on the prevalence of these infections, except for schistosmiasis, in underdeveloped countries. This study aimed to determine the seroprevalence of *Echinococcus multilocularis*, *Schistosoma mansoni,* and *Toxoplasma gondii* antibodies in populations from the Monduli and Babati districts in Tanzania.

**Methods:**

A total of 345 blood samples were collected from 160 and 185 randomly selected households from Babati and Monduli districts, Tanzania between February and May of 2012 and analyzed them using the enzyme linked immunosorbent assay. The antibodies were determined using the NovaLisa® Toxoplasma gondii IgG, NovaLisa® Schistosoma Mansoni IgG, NovaLisa® Echinococcus IgG and NovaLisa® Toxoplasma gondii IgM kits (Novatec, Germany).

**Results:**

The seropositivity estimated for *E. multilocularis*, *S. mansoni*, and *T. gondii* IgG was 11.3% (95% confidence interval (CI): 7.96 - 14.6), 51.3% (95% CI: 46.0 - 56.5), and 57.68% (95% CI: 52.5 - 62.9), respectively. The seropositivity for *T. gondii* IgM was 11.3% (95% CI: 7.96 - 14.6). Living in the Monduli district was found to be the main risk factor for IgG seropositivity for both schistosomiasis (OR =1.94; 95% CI: 1.23 - 3.08; p =0.005) and toxoplasmosis (OR =2.09; 95% CI: 1.31-3.33; p =0.002).

**Conclusions:**

These results suggest that restricting disease transmission, implementing control measures, and introducing training projects to increase public awareness are imperative, particularly for the Monduli district.

## Background

Parasitic infections are common in developing countries due to poor sanitation and inadequate personal hygiene. Undiagnosed and untreated parasitic infections can have severe consequences. Furthermore, many parasitic infections can persist for decades and result in significant morbidity and mortality. Diseases such as echinococcosis, schistosomiasis, and toxoplasmosis are globally widespread, and except for schistosomiasis there is little data on the prevalence in most developing countries, including Tanzania.

Echinococcosis is a zoonotic disease caused by metacestodes of *Echinococcus granulosus*, *E. multilocularis*, *E. vogeli*, and *E .oligarthrus* [1,2]. The larvae invade through the intestines and establish in distinct organs of the body; the liver is the most common organ affected, followed by the brain and muscles [3]. Transmission of *Echinococcus* relies on carnivores feeding on infected herbivores, thus a human infection is usually a dead end for the parasite, as it does not result in further transmission. In humans infected with *Echinococcus,* 60% remain asymptomatic. Infection is confirmed by serological examination, because infected humans do not discharge *Echinococcus* eggs [2,4]. The two most medically relevant species, *E. granulosus* and *E. multicularis,* cause human cystic echnicoccosis (CE) and alveolar echinococcosis, respectively. CE contributes to more than 95% of the 2–3 million cases reported worldwide and 600,000 disability adjusted life years, while human alveolar echinococcosis gives rise to 0.3 - 0.5 million reported cases worldwide [5]. CE due to *E. granulosus* occurs on a global scale and is endemic in the Meditarranean, Central Asia, Northern and Eastern Africa, Australia, and South America [6,7]. The genetically similar *E. multicularis* causes alveolar echinococcosis in the northern hemisphere, specifically in China, Russia, Europe, and North America [8]. The existing data on CE in Africa is insufficient and out of date; therefore a complete picture of the epidemiological situation cannot be achieved.

Schistosomiasis, or bilharzia, caused by flukes of the genus *Schistosoma,* is one of the most common neglected tropical diseases. This parasite infects approximately 240 million people in 74 countries worldwide [9]. More than 90% of these cases are reported in Sub-Saharan Africa, where they have reported 200,000 deaths per year [10]. According to the World Health Organization in 2006, schistosomiasis and soil-transmitted helminthiasis contributes to more than 40% of all the tropical diseases, not including malaria. Furthermore, health statistics report that fewer than 5% of infected individuals are receiving praziquantel, an affordable anti-helminthic drug [11].

*Toxoplasma gondii* is an Apicomplexa parasite that infects a majority of bird and mammal species; however, felines are the only host that excretes the oocyst. Humans can become integrated into the life cycle of the parasite as an intermediate host either congenitally or post-natally by ingesting contaminated food or water, or an infected tissue cyst [12–15]. Congenitally infected individuals may experience neurological and vision defects as a result of infection. In post-natally infected immunocompromised patients *T. gondii* can cause chronic infections with symptoms such as retinochoroiditis, encephalitis, myocarditis, and hepatitis [16,17]. In immunocompetent patients, post-natal infections are usually asymptomatic, however, there can be exceptions that result in severe systemic illness. Globally, more than 2 billion people have been infected with this parasite [18]. Interestingly, the prevalence of *T. gondii* infection varies geographically as 6.7% of the Korean population is infected [19], 12.3% of the Chinese [20], 23.9% of the Nigerian [21], 46% of the Tanzanian [22] and 47% of the population in the underdeveloped areas of France [23].

Echinococcosis, schistosomiasis, and toxoplasmosis pose a serious public health issue worldwide because there are no commercially available vaccines and currently, treatment depends on chemical drugs [24–26]. The objective of the present study was to investigate the *E. multilocularis, S. mansoni,* and *T. gondii* seroprevalence in the populations of the Babati and Monduli districts. The results provide a foundation to execute control strategies against these diseases in this province and elsewhere.

## Methods

### Study area (Babati district)

Babati district is a district of Manyara Region of Tanzania, East Africa. The administrative capital of the district is Babati town, 172 km south of Arusha. The district covers an area of 6,069 km^2^, a large proportion (640 sq km) of which is covered by the water bodies of Lake Babati, Lake Burunge and Lake Manyara. The district is bordered to the north by Arusha Region, to the southeast by Simanjiro District, to the south by Dodoma Region, to the southwest by Hanang District, and to the northwest by Mbulu district.

### Geographical and climatic information

Babati district is located below the Equator between latitude 3° and 4° South and longitude 35° and 36° East. It has two periods of rain, the low rainfall period, which lasts from October to December, and high rainfall period which begins in March and ends in May. The climate zones observed are tropical and temperate with the average temperature ranging between 17-27°C.

### Main income resource and population

About 90% of the population of Babati District live in the rural areas and depend on agriculture and livestock for their livelihood. Mixed crop-livestock, mostly maize-based system are widely found in the district that are intercropped with varying species, such as common beans, pigeon peas and sunflowers, depending on altitude and availability of rainfall.

According to the national census of 2012, the district has a population of 312,392 people. Only 44% of the 96 villages have health facilities, such as dispensaries or health centers, whereas other villages are covered by the mobile and outreach services, especially for mother and childcare.

### Monduli district

Monduli District is one of the five districts of the Arusha Region of Tanzania. It is located in the northeastern section of the country. It is bordered to the north by Kenya, to the east by the Kilimanjaro Region and Arumeru District, to the south by the Manyara Region and to the west by Ngorongoro District and Karatu District. The town of Monduli is the administrative seat of the district. According to the Tanzania National Census of 2012, the population of Monduli District is 158,929 people and the total area of the district is 14,201 km^2^. The rainfall ranges from 500 mm to 1000 mm per year. The district possesses rich wildlife resources that give a potential for tourism activities and acts as a source of income for the country. The people that reside in the Monduli District are the Maasai people, known to be nomads who practice sheepherding as their occupation. The major economic activities are crop production and livestock keeping, however, livestock keeping is more dominant. Monduli District has one hospital, one health centre, and 22 dispensaries.

In parallel with a study of seroprevalence of sparganosis among inhabitants of the two districts (Babati and Monduli); This study was conducted to determine the seroprevalence of *Schistosomia mansoni*, *Echinococcus multilocularis* and *Toxoplasma gondii* antibodies among the inhabitants of the two districts (Babati aand Monduli) in Northern Tanzania for which the prevalence of these diseases has not been reported before in these districts.

A cross-sectional study was carried out among inhabitants in Babati and Monduli districts. The villages which are saved by the health facility were randomly selected. The participants were randomly selected from outpatient in the health center and were grouped according to the villages they belonged to and only one participant was selected from each household to participate in the study. Approximately 4 ml of venous blood was collected from each participant using vacutainers and the serum was pipetted into cryogenic vials which was then stored at −20°C. A total of 345 blood samples were collected from 160 and 185 randomly selected households from Babati and Monduli districts, respectively, from three different wards in each of the districts between February and May of 2012 (Table 1).

**Table 1:** **General characteristics of the participants from Monduli and Babati districts, Tanzania (n =345)**

**Characteristics**	**N (%)**
**Age group (years)**	
1-15	15 (4.3)
16-30	128 (37.1)
31-45	99 (28.7)
46-60	75 (21.7)
61-75	23 (6.7)
76-90	4 (1.2)
>91	1 (0.3)
**Gender**	
Male	136 (39.4)
Female	209 (60.6)
**District**	
Babati	160 (46.4)
Monduli	185 (53.6)
**Ward**	
Mamire	47(13.6)
Galapo	31 (9.0)
Magugu	82 (23.8)
Mto wa mbu	54 (15.7)
Losirwa	103 (29.9)
Esilelia	28 (8.1)

The ethical approval to use these samples specifically for this entire study was obtained from the National Institute for Medical Research (NIMR), an institution under the Ministry of Health and Social Welfare, Tanzania. It is the sole institution in the country, responsible for ethical approval of all research involving human and human material. The National Institute for Medical Research gave ethical approval (Ref: NIMR/HQ/R.8a/VOL.IX/1285) for serological studies on *Spirometra* (unpublished data) and co-infections with echinococcosis, schistosomiasis and toxoplasmosis, from the 4 ml of blood taken from each volunteer. Respective health and government authorities at regional and district level received copies of the ethical approval as required for the implementation of the study. At the individual level, a written consent was sought after detailed explanation of the purpose and the benefits of the study. Participants were informed that their participation was totally voluntary and that they were free to participate or not to participate to the study without giving any reason whatsoever. Written and signed or thumb-printed consents were obtained from all participants before were involved in the study. In case of the minors or children involved in the study, the next of kin, carer or guardian on behalf signed or thumb-printed the consent form. The samples were sent to the Department of Parasitology, Department of Medicine, University of Malaya, Malaysia in April 2013.

### Serology

The NovaLisa® *Toxoplasma gondii* IgG, NovaLisa® Schistosoma Mansoni IgG, NovaLisa® Echinococcus IgG and NovaLisa® *Toxoplasma gondii* IgM kits (Novatec, Germany) were used in this study. The sensitivity of the respective kits is as follows: 96.6%, 87%, 95% and 95.8% whereas the specificity of aforementioned kits is 98.2%, 95%, 95% and 98%. The analysis was performed in Department of Parasitology, University of Malaya. Statistical analysis was performed using SPSS v.20 (SPSS IBM, New York, U.S.A). A Pearson’s Chi-square (χ^**2**^) test was used to test the association between categorical variables and Post –Hoc analysis was performed on statistically significant variables to explore the association between the variables of interest. Odd ratios (ORs), relative risk (RR) and 95% confidence interval (95% CI) were calculated, furthermore, logistic regression was used to identify the significant predictors and to control the potential confounding effect. A. The level of statistical significance was set at p <0.05 for each test. Prior to analyzing, the data was stratified according to districts (Babati, Monduli), wards (Mamire, Galapo, Magugu, Mtowambu, Losirwa, Esilelia), gender (male/female) and age group (1–15, 16–30, 31–45, 46–60, 61–75, 76–90, >91) in years.

## Results

The general characteristics of participants according to age, gender, district and divisions are shown in Table 1.

### Seroprevalence of echinococcosis

Among the 345 serum samples tested, 39 (11.3%, 95% CI =7.96-14.6) were positive for *Echinococcus multilocularis* antibodies. Univariate analysis was performed to check the association of positive results with gender, age group and district (Table 2). There was no statistically significant association detected with either one of the variables being tested. However, association tested among the wards of both districts (Table 3) showed that there was a significant difference between the wards of Babati district (p value <0.01). Post-hoc analysis showed that there is a significant difference between Mamire and Gelapo (p =0.01, 95% CI 0.101-0.473) as well as Mamire and Magugu (p =0.03, 95% CI =0.0774-0.439). Logistic regression analysis could not detect any significant association between echinococcosis- positive IgG and the examined variables (Table 4). The chi-square results for the distribution of positive IgG and IgM (only *T. gondii*) results according to age groups are shown in Table 5.

**Table 2:** **Univariate analysis for the association between risk factors and seroprevalence**

**Risk factor**	**Echinococcosis**	**Schistosomiasis**	**Toxoplasmosis IgG**	**Toxoplasmosis IgM**
	**N/Total (%)**			
**Gender**				
Male	19/136 (13.9)	65/136 (47.8)	71/136 (52.2)	17/136 (12.5)
Female	20/209 (9.56)	112/209 (53.6)	128/209 (61.2)	22/209 (10.5)
RR	1.46	0.892	0.852	1.19
(95% CI)	(0.810-2.633)	(0.718-1.107)	(0.702-1.035)	(0.655-2.153)
P-value	0.207	0.293	0.097	0.572
**District**				
Babati	18/185 (9.73)	109/185 (58.9)	123/185 (66.5)	16/185 (8.65)
Monduli	21/160 (13.1)	68/160 (42.5)	76/160 (47.5)	23/160 (14.4)
RR	0.962	1.400	1.567	0.937
(95% CI)	(0.891-1.04)	(1.13-1.74)	(1.22-2.01)	(0.867-1.01)
P-value	0.321	0.002	0.000	0.094
**Age group**				
≤60	35/317 (11.0)	164/317 (51.7)	187/317 (59.0)	36/317 (11.3)
>60	4/28 (13.3)	13/28 (46.4)	12/28 (42.8)	3/28 (10.7)
RR	0.773	1.114	1.376	1.060
(95% CI)	(0.296-2.02)	(0.738-1.68)	(0.889-2.13)	(0.348-3.23)
P-value	0.603	0.590	0.098	0.918

**Table 3:** **Univariate analysis for the association between the divisions of districts and seroprevalence**

**Location**	**Echinococcosis**	**Schistosomiasis**	**Toxoplasmosis IgG**	**Toxoplasmosis IgM**
	**N/Total (%)**			
**Babati wards**				
Mamire	15/47 (31.9)	26/47 (55.31)	22/47 (46.8)	8/47 (17.0)
Galapo	1/31 (3.22)	12/31 (38.7)	10/31 (32.2)	6/31 (19.3)
Magugu	5/82 (6.09)	30/82 (36.5)	44/82 (53.6)	9/82 (10.9)
P-value	0.000	0.106	0.127	0.441
**Monduli wards**				
Mto wa Mbu	7/54 (12.9)	23/54 (42.6)	37/54 (68.5)	2/54 (3.70)
Losirwa	10/102 (9.80)	71/102 (69.6)	62/102 (60.8)	10/102 (9.80)
Esilelia	1/29 (3.44)	15/29 (51.7)	24/29 (82.7)	4/29 (13.8)
P-value	0.382	0.03	0.081	0.248

**Table 4:** **Multivariate analysis for the association between risk factors and seroprevalence**

**Risk factor**	**Echinococcosis**	**Schistosomiasis**	**Toxoplasmosis IgG**	**Toxoplasmosis IgM**
	**Adjusted OR (95% CI)**			
**Gender**				
Male/Female	1.40 (0.673-2.927)	1.01 (0.627-1.639)	0.958 (0.589-1.557)	1.02 (0.487-2.122)
P-value	0.365	0.957	0.861	0.966
**District**				
Babati/Monduli	0.811 (0.395-1.662)	1.94 (1.227-3.078)	2.09 (1.310-3.326)	0.557 (0.271-1.148)
P-value	0.567	0.005	0.002	0.113
**Age**				
≤60/>60	0.915 (0.287-2.910)	1.04 (0.462-2.346)	1.56 (0.689-3.551)	1.25 (0.345-4.543)
P-value	0.880	0.923	0.285	0.732

**Table 5:** **Chi-square for age groups**

**Age groups (years)**	**N (%)**	**Echinococcosis**	**Schistosomiasis**	**Toxoplasmosis IgG**	**Toxoplasmosis IgM**
1-10	9 (2.6)	1 (0.3)	4 (1.2)	8 (2.3)	0 (0.0)
11-20	54 (15.7)	1 (0.3)	32 (9.3)	35 (10.1)	6 (1.7)
21-30	80 (23.2)	10 (2.9)	45 (13.0)	48 (13.9)	9 (2.6)
31-40	70 (20.3)	9 (2.6)	33 (9.6)	39 (11.3)	6 (1.7)
41-50	63 (18.3)	10 (2.9)	32 (9.3)	37 (10.7)	8 (2.3)
51-60	41 (11.9)	4 (1.2)	18 (5.2)	20 (5.8)	7 (2.0)
>60	28 (8.1)	4 (1.2)	13 (3.8)	12 (3.5)	3 (0.9)
Total (%)	345(100)	39 (11.3)	177 (51.3)	199 (57.7)	39 (11.3)
*χ* ^*2*^		6.75	3.98	8.88	3.16
P-value		0.344	0.680	0.180	0.788

### Seroprevalence of schistosomiasis

ELISA results showed 177 (51.3%, 95% CI =46.0-56.5) positive samples out of 345 serum samples for *Schistosoma mansoni* antibodies. Statistical analysis (Table 2) demonstrated that a significantly higher prevalence of schistosomiasis in Monduli as compared to Babati (RR =1.40, 95% CI =1.13-1.74). Univariate tests between the wards of Monduli (Table 3) revealed that there is a significant difference in the prevalence of schistosomiasis between the divisions (p =0.03). Post-Hoc analysis showed significant difference among two of the divisions, Losirwa and Mto Wa Mbu (p =0.004, 95% CI =0.0714-0.486). Binary logistic regression also yielded the same pattern of results for districts (OR =1.94, 95% CI =1.23-3.08) and for the other two variables (Table 4).

### Seroprevalence of toxoplasmosis

Out of the 345 serum samples, 199 (57.68%, 95% CI =52.5-62.9) were positive for *Toxoplasma gondii* IgG antibodies. A Pearson’s Chi-square (χ^**2**^) test (Table 2) showed a statistical significant difference between the occurrence of infection among the two districts (RR =1.57, 95% CI =1.22-2.01). Multivariate analysis also indicated that district was the most important factor, with the samples from Monduli having higher seropositivity (OR =2.09, 95% CI =1.31-3.32). As for *T. gondii* IgM, only 39(11.3%, 95% CI =7.96-14.6) serum samples were positive. Univariate (Table 2) and multivariate analysis (Table 4) did not give any statistically significant results for any of the variables being tested.

## Discussion

The antibody detection methods are not 100% specific and sensitive, thus the findings of this study should be interpreted with caution. Furthermore, the specificity and sensitivity of the detection methods depend on the antigen used when performing ELISA. The use of clinical diagnosis for positive cases would have elucidated even more information regarding the prevalence of these infectious diseases. However, this could not be accomplished in this study due to lack of manpower and the required expertise in the sampling site to carry out clinical test. Despite this, the pattern observed helps to access the prevalence in a population.

We detected 11.5% (8.00 - 14.6) seroprevalence among the samples tested. The antigens used cannot differentiate between *E. granulosa* and *E. multilocularis* antibodies. However, because alveolar echinococcosis is prevalent in the Northern hemisphere and cystic echinococcosis is endemic in Sub-Saharan Africa [7], *E. granulosa* could be responsible for the seroprevalence detected but this should be confirmed with clinical tests. Our result reveals a far greater infection rate than the 0.007% reported in Burkina Faso [27], 0.3 - 0.8% in central Sudan [28], and the 2% and 3.5% among Bouya and Topasa populations, respectively, in southern Sudan [29]. The only two countries that are well researched are Kenya and Sudan. In Nigeria, sonar scan results depicted a prevalence of 5.1% [30]. The IgG antibodies can be detected in patients that are currently in the asymptomatic phase [31], which could be the reason for the high seroprevalence observed in this study. Additionally, specificity issues in the enzyme linked immunosorbent assay (ELISA) can lead to cross-reactions with other species of *Echinococcus* or with cysticercosis and give false positive results [1]. Finally, IgG antibody production is dependent on many factors such as size, number, surface antigen orientation, and the condition of the hydatid cysts [2]. No antibody production has been observed due to the presence of small cysts, intact cysts, or calcified cysts [32].

The incidence of cystic echinococcosis is not uniformly distributed in Sub-Saharan Africa. Overall, rates of infection are higher in the arid regions of Sahel and in eastern and southern Africa where the household income primarily depends on livestock husbandry. Rates of infection are less frequent in areas with adequate rainfall in western and central Africa where livestock is limited [7]. The difference in infection rates could be due to the fact that the governing bodies place low importance on the dry pastoral areas because they contribute little to the gross domestic product. Sub-Saharan Africa is at the bottom of the list in terms of contribution to world economics, therefore, the majority of the area is underdeveloped, and rural areas are a significant risk factor for cystic echinococcosis [2,33]. Age can be another risk factor [33,34], but in the present study age did not have a significant effect. Another risk factor for echinococossis is a high prevalence of hydatidosis in livestock and not de-worming the animals [35]. One of the main occupations in Babati and Monduli is livestock keeping, which may also increase their risk of disease [36,37]. We observed a significant difference in the seroprevalence among the Babati wards which could be due to unhygienic conditions, genetic predisposition, malnutrition-related immunosuppression [38], close contact with dogs, and a high number of infected livestock.

*Schistosoma haemotobium* and *S. mansoni* cause urogenital schistosomiasis and intestinal schistosomiasis, respectively, and in Africa they contribute more to mortality rate than other species [39]. Globally, the prevalence of these parasites tends to increase due to man-made lakes, construction of dams, and irrigation projects. Civil wars also increase prevalence, because they are one of the primary reasons for migration [39,40]. Tanzania has the second highest prevalence of schistosomiasis in Africa, coming just behind Nigeria [39,41]. The water supply projects initiated in the late 1970’s have been blamed for the transmission of the parasite because they provide a suitable habitat for the intermediate host, snails, which promote the spread of disease [42–46]. *Schistosoma mansoni* are rarely present in the coastal regions because of thermal exclusion and because there is no intermediate host [47]. The potential host candidates for S. mansoni are *Biomphalaria choanomphala*, *B. pfeifferi*, *B. sudanica,* and *B. angulosa* [39,48]. *Biompharlaria choanomphala* is confined to large bodies of water and hydro-electrical dams in the south and east-south of the country, whereas *B. sudanica* is found in dams, on the southern shores of Lake Victoria, and in the north [42]. *Biomphalaria pfeifferi* is widely distributed in seasonal water sources and swamps throughout the majority of Africa, whereas *B. angulosa* is primarily observed in swamps only in the southern highlands [39,47,49]. In 2012, the prevalence of schistosomiasis was estimated to be 51.5% [50]. The present study confirms this estimate (51.3%). A large number of intermediate hosts may explain the high seroprevalence observed in this study.

In *S. mansoni* endemic places, once the immigrants are exposed to the parasite, they also tend to show the same age-pattern as of the residents in terms of infection [51]. Children under 15 years old are the most prone to *S. mansoni* infection, but it does affect people of all age groups [47,48]. The high infection rate in children under 15 could be due to a more frequent contact with water, or it could be due to the development of immunity in adults that lowers prevalence in older populations [52]. However, our results can be explained by high occupational contact or the use of contaminated water for household work [53]. We observed a significant difference between the districts and between the Monduli wards (Mto wa Mbu and Losirwa), which suggests that there is a significantly higher infection rate in the Monduli district compared to Babati. In regards to the wards, our results reveal a higher seroprevalence in the Losirwa ward than in Mto wa Mbu. Several socio-epidemiological factors such as population movement, distance from origin of disease, urbanization, employment status, cleanliness, and the level of fecal contamination in the water supply could explain the high transmission of schistosomiasis in these areas. There is also an increased susceptibility to infection if one handles snails or practices fishing, farming, bathing, and swimming in contaminated water bodies.

Over the course of a *T. gondii* infection, IgM antibodies are typically the first class of antibodies present in primary response, and they also subside more quickly than IgG antibodies [54]. The most common serological marker to diagnose a recently acquired infection is to measure the specific IgM antibodies [55]. In this study, the seroprevalence of *T. gondii* IgM antibodies was 11.3%, which demonstrates that most infections are not acute, and thus the IgM antibody titer is too low to be detected. Another way to distinguish between an acute and a past infection is by using the IgG avidity test. The most convenient and widely used methods to diagnose *T. gondii* infection is by measuring the IgG antibodies through Sabin-Feldman dye test, ELISA, Indirect immunofluorescent assay (IFA) and the modified direct agglutination test [56].

The seroprevalence of *T. gondii* IgG antibodies in this study demonstrate that the residents of Monduli and Babati experience a high exposure to *T. gondii*. Our study found 57.7% prevalence of *T. gondii*, which is higher than a study carried out in the Tanga district that reported a seroprevalence of 45.7% [22]. Other Tanzanian studies report 35.0% prevalence in Dar es Salaam [57], and a high seroprevalence of 72.2% was reported among the Hadzabe in the northern region of the country [58]. The seroprevalences reported by the present study are similar to several studies performed on exposed groups in Africa. For example, seroprevalences of 52.4% and 42.6% were reported among slaughterhouse workers in Egypt and Djibout, respectively [59,60]. Similarly, studies in Congo yielded 41.9% seroprevalence in cattle breeders and slaughterhouse employees [61], whereas in Gezira Province, Sudan, 41.7% of pregnant women were seropositive [62].

Our findings did not concur with patterns of reduced seroprevalence among older populations that had been observed in studies carried out in Nigeria and Congo [61,63]. This trend was not observed in this study, which could be due behavioral differences in the societies. We found no statistical difference in seroprevalence between males and females (Table 2), however other studies have suggested that gender does influence exposure or immune response towards *T. gondii* [61,64]. The lack of difference in the antibody count between the two genders in our study could be due to similar everyday activities in which the individual is exposed to the parasite, which as a result does not make one gender more prone to the parasite that the other. We observed a difference in seroprevalence between the Monduli and Babati districts, which implies that factors that influence the high infection rate in Monduli are not present in the Babati district. These factors may include consumption of contaminated water, fruits, and undercooked meat [65–67]. Eating raw and undercooked meat is still practiced in some areas of Tanzania [68].

## Conclusion

The lack of sufficient information regarding zoonotic disease could be a result of ineffective public education and a lack of communication between veterinary and human health professionals. Insufficient information prevents the people from taking precautionary measures, which in turn lead to an increased risk of infection. In future studies, the surveys used should have more depth to gain information regarding the socioeconomic status of the individuals being used in the study. Our findings show that there is a higher percentage of *S. mansoni* and *T. gondii* infections in the Monduli district as compared to the Babati district. In terms of the wards of the Monduli district, Losirwa has a higher risk of infection to the *S. mansoni* parasite as opposed to the Mto Wa Mbu ward. This suggests that there is a pressing need to improve education about the risk factors and prevention methods, especially in the Monduli district, which could result in a significant reduction in disease transmission.
